# Experience With Extended Follow-Up of Prophylactically Implanted Defibrillators for High-Risk Patients With Hypertrophic Cardiomyopathy

**DOI:** 10.1016/j.jacadv.2024.101317

**Published:** 2024-10-09

**Authors:** Barry J. Maron, Susan Casey, Jay D. Sengupta, Scott W. Sharkey, Martin S. Maron, Ethan J. Rowin

**Affiliations:** a Hypertrophic Cardiomyopathy Center, Lahey Hospital and Medical Center, Burlington, Massachusetts, USA; b Joseph F. Novogratz Family Heart Rhythm Science Center, Minneapolis Heart Institute Foundation, Minneapolis, Minnesota, USA

**Keywords:** hypertrophic cardiomyopathy, implanted defibrillators, sudden death

**Figure:**
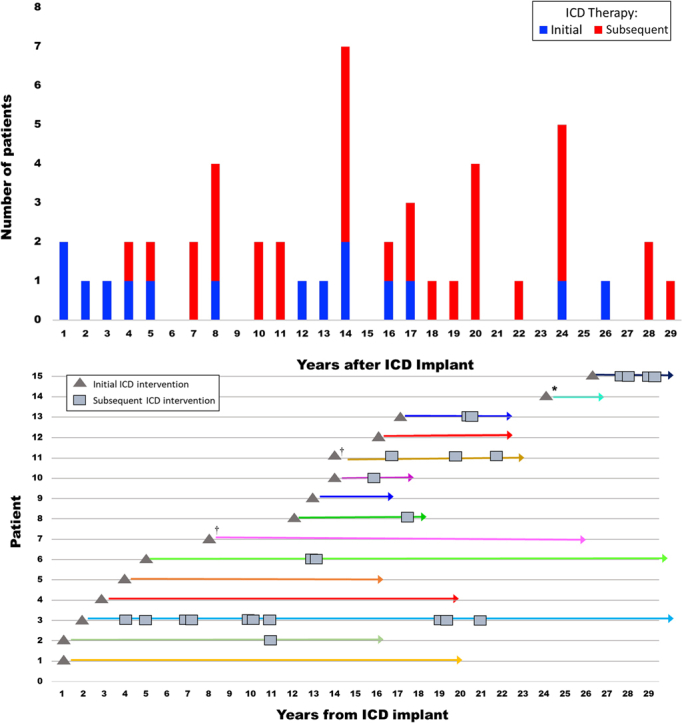

Implantable cardioverter-defibrillators (ICDs) have proved highly effective for preventing arrhythmic sudden death (SD) in patients with hypertrophic cardiomyopathy (HCM). This paradigm management change is in part responsible for marked reduction in SD events and overall disease-related mortality.^1^^,^^2^ However, outcomes related to ICD performance over long postimplant periods in this disease remain incomplete. We have accessed here a unique ICD cohort with the longest time following device placement, as the earliest systematic ICD initiative to prevent SD in HCM.

The ICD study cohort comprises 56 patients from Minneapolis Heart Institute.^2^ Implants began in 1992, preceding by 8 years the initial ICD report in HCM,^3^ 11 years before the introduction of HCM-ICD therapy to consensus guidelines.^4^ Study patients were those followed prospectively at this institution, ≥15 years after implant until April 2024 (range 31 years; mean 20 ± 4 years). Appropriate ICD therapies were recorded as time elapsed postimplant.

Patients considered at increased SD risk received ICDs based on ≥1 major risk markers according to American Heart Association/American College of Cardiology guidelines and/or accepted HCM practice patterns.^2^^,^^5^

Risk factors prompting primary prevention implants were: family history HCM-SD (n = 18; 32%); repetitive and/or prolonged nonsustained ventricular tachycardia (VT) on ambulatory monitor (n = 18; 32%); recent syncope (n = 12; 21%); massive left ventricular (LV) hypertrophy ≥30 mm (n = 11; 20%); LV apical aneurysm (n = 2; 4%); and extensive LV late gadolinium enhancement (n = 3; 6%). No patient had end-stage systolic dysfunction (mean ejection fraction, 64%; range 55% to 80%).

Continuous and categorical data are expressed as mean ± SD, median, or n (%). Subgroups were compared with an unpaired Student’s t-test, chi-square test, or Fisher’s exact test where appropriate.

Fifty-six patients (66% male) were implanted for primary (n = 50) or secondary (n = 6) SD prevention at 39 ± 15 years (range 13-69 years), followed by 20 ± 4 years (range 15-31 years). LV thickness was 24 ± 6 mm (range 15-45 mm).

Fifteen patients (27%) experienced 48 interventions terminating ventricular fibrillation (n = 15) or rapid VT (n = 32) and 1 unresolved. Of the 15, 11 were asymptomatic, and 4 had mild heart failure symptoms. Initial ICD interventions occurred at 50 ± 16 years (range 15-70 years), 13 years after implant (range 1-26 years), and ≥12 years in 8 patients. The most common risk markers were family history of HCM-SD, nonsustained VT (3 patients each), and massive hypertrophy (n = 2).

Of the 15 patients, 10 experienced additional appropriate ICD interventions following initial therapy (N = 33). Number of subsequent device interventions were: 1 intervention (n = 5), 2 interventions (n = 3), 3 interventions (n = 2), 4 interventions (n = 2), 5 interventions (n = 1), 7 interventions (n = 11), up to 11 interventions (n = 1; mean number = 3) (Figure 1).

**Figure 1:**
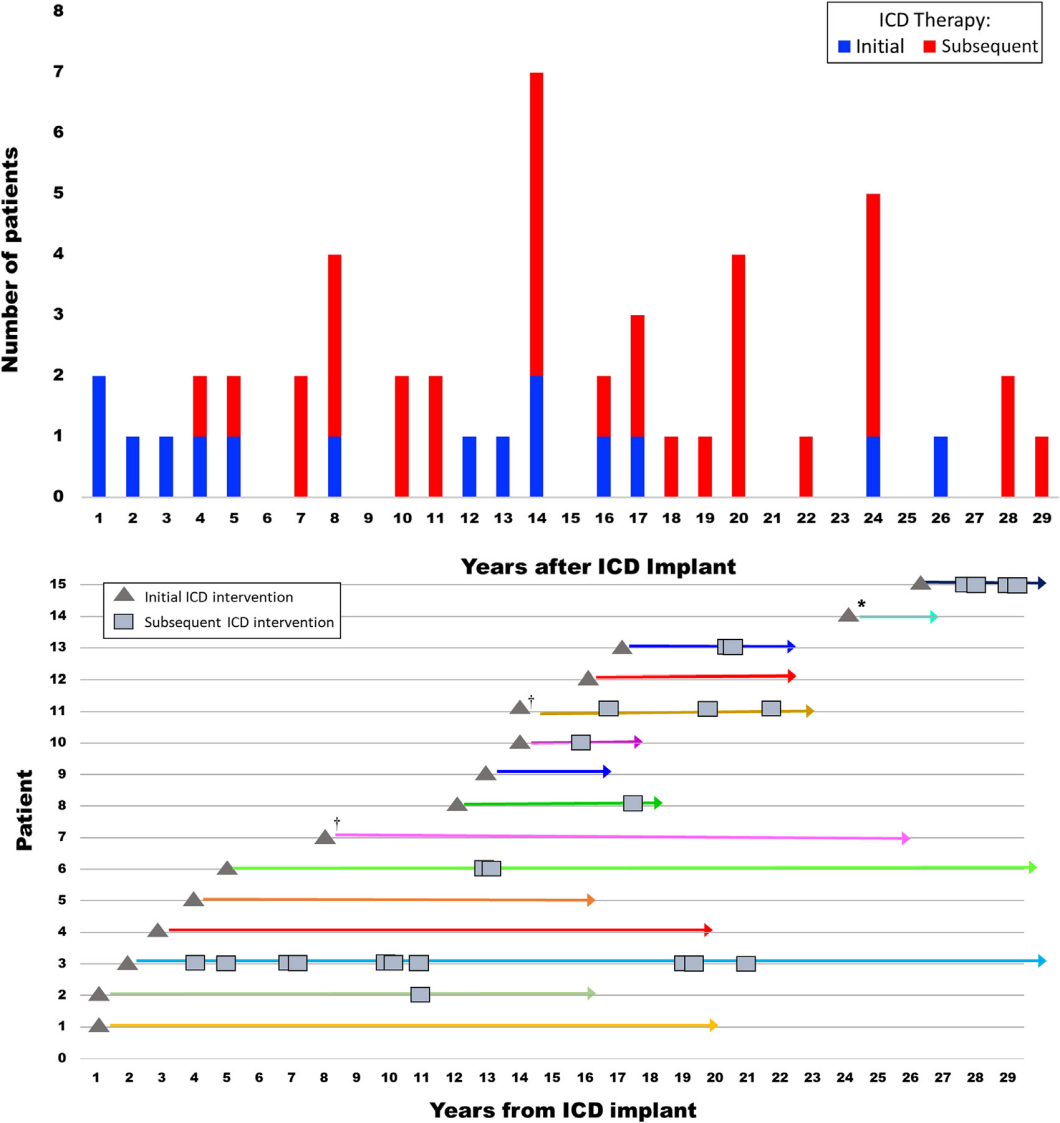
Long-Term Appropriate ICD Therapy in HCM Long-term appropriate ICD therapy in HCM. (Top) The 48 appropriate ICD therapies in 15 high-risk HCM patients who were followed postimplant for an average of 20 years (up to 31 years). ICD events are plotted with respect to delay following implant. Initial (I) therapies are shown in blue and subsequent therapies (S) in red. (Bottom) Showing for each patient initial (n = 15) and subsequent (n = 33) interventions and dormancy periods without therapies. ∗5 interventions in 1 year; †4 interventions in 1 year. HCM = hypertrophic cardiomyopathy; ICD = implantable cardioverter-defibrillator.

The 33 subsequent ICD interventions occurred 4 to 29 years after ICD implant (median 16 years). Periods of device dormancy (ie, absent VT/ventricular fibrillation interventions) ranged from 1 to 26 years (mean 6 years): ≥5 years in 10 and ≥8 years in 9.

Of the 15 patients with ICD therapy, 14 (93%) have survived to 61 ± 12 years (range to 78 years) 1 died with multiple comorbidities including hepatic cirrhosis and lymphoma. There were no differences in baseline characteristics between 15 patients with appropriate ICD therapy and 41 patients without such interventions: implant age, sex, risk markers, LV thickness, and outflow obstruction (*P* > 0.05 for each).

Ten of 56 study patients experienced ≥1 inappropriate shocks from atrial fibrillation or supraventricular tachycardia; 6 had fractured/failed leads requiring extraction. Six patients experienced generator or lead recalls, and 1 patient had pocket infection requiring system extraction.

The primary prevention ICD paradigm in HCM has evolved over 25 years, beneficially altering clinical course for many vulnerable patients by preventing arrhythmic SDs.^1^^,^^2^^,^^5^ ICDs have been responsible in part for an important decrease in overall HCM-related mortality to ≤0.5%/year (0.3%/year for SDs).^2^

HCM patients considered candidates for SD prevention are usually young and obligated to living decades with implanted devices. Understandably, a major question from patients is whether/or when they will experience unpredictable device interventions.

These data extend our observations in high-risk HCM patients by employing a unique study design providing extended follow-up, made possible by a pioneering ICD program begun in the early 1990s.^3^ We are therefore able to report outcomes over average follow-up of 20 years, now extending >30 years. Notably, 48 appropriate ICD therapies were identified, likely representing aborted SD events in young predominantly asymptomatic HCM patients, demonstrating how ICDs can change clinical course of this complex disease.^1^, ^2^, ^3^ Indeed, >50% of our cohort experienced initial intervention ≥12 years postimplant.

We identified a subset of patients who experienced initial device therapy >20 years (up to 29 years) after implant. One received an ICD prophylactically after his son’s HCM-SD; 17 years later (age 67 years), he unexpectedly experienced his own ICD therapy, followed 3 years later by 2 more interventions. Another asymptomatic obstructive HCM patient was implanted for primary prevention at age 29 years due to HCM-related SD in his father. Initial device therapy terminating VT was delayed 26 years until age 55 years, followed by 3 subsequent interventions.

In conclusion, a substantial proportion of high-risk HCM patients experienced appropriate ICD therapies over extended postimplant periods, demonstrating the under-recognized capacity for particularly delayed interventions aborting SDs over 3 decades. These novel observations enhance understanding and expectations for ICD performance, including frequency and timing of device therapies. These data argue against clinical inclinations to remove ICDs as unnecessary after prolonged device dormancy.

## Funding support and author disclosures

Dr M. Maron serves as a consultant for Cytokinetics, iRhythm (with grant), Imbria Pharmaceuticals, and TAKEDA Pharmaceuticals (with grant). Dr Rowin serves as a consultant for Cytokinetics and iRhythm. All other authors have reported that they have no relationships relevant to the contents of this paper to disclose.
